# Factors associated with common mental disorders, suicidal risk, and stimulant use in college students

**DOI:** 10.1590/0034-7167-2024-0501

**Published:** 2025-12-08

**Authors:** Gisele Carolina Bianchi, Adriana Inocenti Miasso

**Affiliations:** I Universidade de São Paulo. Ribeirão Preto, São Paulo, Brazil

**Keywords:** Students, Universities, Mental Disorders, Suicide, Psychotropic Drugs., Estudiantes, Universidades, Trastornos Mentales, Suicidio, Psicotrópicos.

## Abstract

**Objectives:**

to identify factors associated with common mental disorders, suicide risk, and stimulant use among undergraduate students.

**Methods:**

a quantitative, cross-sectional, correlational, and analytical study was conducted with a sample of 190 students from a public university. Validated instruments were used for data collection, and logistic regression models were used for analysis.

**Results:**

71.6% of participants tested positive for common mental disorders. Suffering losses during the pandemic was a risk factor for common mental disorders, and feeling satisfied with their life when accessing social media was a protective factor. It was found that 23.9% were at high risk for suicide, with a higher risk for those without religion, using non-prescription medications, diagnosed with a mental disorder, and consuming alcohol. Furthermore, 73.7% of participants used stimulants. Stimulant use showed no significant relationship with the variables analyzed.

**Conclusions:**

this research contributes evidence for promotion and prevention actions in academic mental health.

## RESUMEN

**Objetivos:**

identificar los factores asociados con trastornos mentales comunes, riesgo de suicidio y consumo de estimulantes en estudiantes universitarios.

**Métodos:**

se realizó un estudio cuantitativo, transversal, correlacional y analítico con una muestra de 190 estudiantes de una universidad pública. Se utilizaron instrumentos validados para la recopilación de datos y modelos de regresión logística para el análisis.

**Resultados:**

el 71,6 % de los participantes dio positivo en la prueba de trastornos mentales comunes. Sufrir pérdidas durante la pandemia fue un factor de riesgo para los trastornos mentales comunes, y sentirse satisfecho con la vida al acceder a las redes sociales fue un factor protector. Se encontró que el 23,9% presentaba un alto riesgo de suicidio, con mayor riesgo en quienes no profesaban religión, consumían medicamentos sin receta, tenían un trastorno mental diagnosticado y consumían alcohol. Además, el 73,7% de los participantes consumía estimulantes. El consumo de estimulantes no mostró una relación significativa con las variables analizadas.

**Conclusiones:**

esta investigación aporta evidencia para la promoción y prevención de la salud mental académica.

## INTRODUCTION

Entering university can predispose students to greater vulnerability to impatience, irritability, anxiety, and stress, with the potential for mental disorders^(1)^. The COVID-19 pandemic has altered academic dynamics, with the suspension of in-person activities, internships, and the adoption of remote learning, impacting the quality of student education^(2)^. These changes increased feelings of anguish among undergraduate students as well as anxiety and depressive symptoms^(3)^.

Research reveals 69.8% prevalence of common mental disorders (CMDs) among undergraduate students after the start of the pandemic^(4)^.

CMDs correspond to a set of non-psychotic symptoms, such as anxiety, depression, insomnia, fatigue, body aches, irritability, memory lapses, difficulty concentrating and nonspecific somatic manifestations, which cause psychological distress and functional impairment, but do not fully meet the diagnostic criteria for severe mental disorders^(5)^.

A study suggests that the development of CMDs in students may be associated with a sedentary lifestyle, tobacco use, and use of substances believed to improve cognitive performance, as well as feelings of sadness, anhedonia, anxiety, depressive symptoms, and suicidal ideation^(6)^.

Suicidal risk can be understood as the possibility that self-destructive thoughts evolve into concrete actions, resulting in self-inflicted death^(7)^. This is a complex phenomenon, influenced by a variety of determinants, such as sociodemographic factors, economic conditions, and health-related aspects, which affect both the general population and more vulnerable groups^(8)^, such as undergraduate students. Studies show that the presence of previous manifestations of psychological distress, a common condition among undergraduate students, significantly contributes to greater susceptibility to suicidal behavior^(9,10)^.

Given the intense cognitive demands and high levels of stress experienced in the university environment, undergraduate students may resort to various strategies to minimize emotional overload. Among these strategies, the use of stimulant substances stands out, which act on the central nervous system, promoting increased alertness, improved concentration, increased motivation, and, in some cases, antidepressant effects. These substances are often associated with potential improvements in cognitive performance and mood regulation^(11)^.

A survey of undergraduate students revealed that 53.7% of participants reported using stimulants, with methylphenidate hydrochloride, energy drinks, and amphetamines being the most frequently mentioned. The main reasons given for this use included improving cognitive abilities and achieving faster and more effective learning^(12)^.

Prolonged use of stimulants by undergraduate students has been associated with several adverse effects, some of which may be permanent. Among the identified harms is the potential for worsening anxiety states, initially physiological, which can evolve into more severe pathological conditions, including an increased risk of suicidal behavior. The lack of knowledge about the use of these substances and the associated risks constitutes a significant knowledge gap^(13)^.

Despite the relevance of CMDs, stimulant use, and suicide risk among undergraduate students, there is a gap in the literature regarding the simultaneous investigation of these outcomes in samples from public higher education institutions. Deepening the understanding of the factors that contribute to these conditions is essential to inform more effective support strategies and specific interventions aimed at alleviating student psychological distress and fostering a healthier, more welcoming, and sustainable academic environment.

## OBJECTIVES

To investigate the factors associated with CMDs, suicide risk, and stimulant use among students at a public university in the state of São Paulo, Brazil.

## METHODS

### Ethical aspects

This study was approved by the Research Ethics Committee (Certificate of Submission for Ethical Consideration 55125921.2.0000.5393), following the guidelines established by Resolution 466 of December 12, 2012 of the Brazilian National Health Council, which regulates research involving human beings. All participants signed the Informed Consent Form (ICF).

This article is derived from a master’s thesis entitled “*Transtornos mentais comuns, risco de suicídio e uso de estimulantes em estudantes de uma universidade pública*”^(14)^.

### Study design, period and location

This is a quantitative, cross-sectional, correlational-analytical study conducted from March to July 2023 at higher education institutions located on the *campus* of a public university in the state of São Paulo, Brazil. The research was based on the STrengthening the Reporting of OBservational studies in Epidemiology tool.

### Population and sample: inclusion and exclusion criteria

The convenience sample comprised 190 students enrolled in undergraduate programs at the university *campus* under study. Students aged 18 or older and regularly enrolled in undergraduate programs at the institution were included. Those who completed the instrument incompletely were excluded, thus hindering the analysis of the study’s three outcome variables: CMD, suicide risk, and stimulant use.

### Study protocol

For data collection, an email was sent to all Undergraduate Committees of the units on the *campus* under study, requesting that the invitation letter be forwarded to students. Thus, all undergraduate students received a formal individual invitation from the Undergraduate Committees via institutional email. The letter presented the research title, its objective, the rationale, and the potential contributions of the study, including a link to access the Google Forms^®^ platform at the end.

Students who agreed to participate in the research accessed the provided link, where they found the ICF and data collection instruments. After agreeing and electronically signing the ICF, they were directed to complete the questionnaires. The response period was set at 60 days from the date the invitation letter was sent, after which receipt of the instruments officially closed.

Three instruments were used for data collection: 1 - Questionnaire prepared by the researchers, covering sociodemographic, economic, academic information, health history and stimulant use, which underwent form and content assessment by experts in the field; 2 - Self-Reporting Questionnaire-20, containing 20 questions for screening CMD, validated for use in Brazil^(15)^. In this study, the cut-off points established in the validation were adopted: positive case for women who indicated “yes” in eight or more items, and for men, with six or more affirmative responses; 3 - Mini International Neuropsychiatric Interview (MINI) - a brief diagnostic instrument aligned with the criteria of the DSM-III-R/IV and the International Classification of Diseases, 10^th^ revision, recognized for its reliability and international validity^(16)^. The instrument is organized into independent diagnostic modules, and responses consist of a dichotomous variable of “yes” or “no”^(16)^. Organized into independent diagnostic modules, the MINI uses dichotomous responses (yes/no), and for the purposes of this study, module C, referring to suicide risk, was specifically applied.

### Analysis of results and statistics

Data analysis was performed using a quantitative approach. Responses obtained through the Google Forms^®^ platform were exported to a spreadsheet and later transferred to the Statistical Package for the Social Sciences version 21.5.

Initially, univariate analyses were performed using the chi-square test. In cases where the categories had a frequency of fewer than five participants, Fisher’s exact test was applied. To assess the influence of independent variables on study outcomes, multivariate logistic regression models were developed. Independent variables that presented a p-value less than 0.05 in the univariate analysis were included in the model. Associations with p<0.05 were considered statistically significant.

## RESULTS

A total of 190 undergraduate students participated in this study. The majority of participants reported not having children (98.4%), not having a partner (92.5%), being from the state of São Paulo (82.6%), being white (77.5%), being female (71.8%), being between 20 and 24 years old (63.2%) and having heterosexual orientation (51.6%).

It was found that 71.6% of participants tested positive for CMDs. The logistic regression model showed that participants who reported having suffered some harm during the pandemic were 10.278 times more likely to test positive for CMDs (OR=10.278; CI=2.301-57.446; p=0.004). Feeling satisfied with one’s life when using social media proved to be a protective factor against CMDs (OR=0.071; CI=0.013-0.286; p=0.001) (Table 1).

**Table 1:** Logistic regression model adjusted for common mental disorders in undergraduate students from different teaching units of a public university (N=190), Ribeirão Preto, São Paulo, Brazil, 2023

Variables		Coefficient	Standard error	Value *p*	OR^*^	95%CI^*^ (OR)
Intercept	-----	2.115	1.145	0.065	8.287	0.959 - 91.298
Suffered any harm during the pandemic	Yes	2.330	0.807	0.004	10.278	2.301 - 57.446
	No	------	------	------	------	
Tested positive (COVID-19)	Yes	-1.142	0.695	0.100	0.319	0.075 - 1.191
	No	------	------	------	------	
Has a history of illness	Yes	0.124	0.857	0.885	1.132	0.202 - 6.081
	No	------	------	------	------	
Feels satisfied with life when accessing social networks	Yes	-2.642	0.777	0.001	0.071	0.013 - 0.286
	No	------	------	------	------	
Has a diagnosis of a mental disorder	Yes	0.680	1.097	0.535	1.975	0.230 - 18.152
	No	------	------	------	------	
Has a diagnosis of mental disorder	Yes	-0.026	0.917	0.977	0.974	0.160 - 6.200
	No	------	------	------	------	
Uses tobacco (POD)	Yes	-2.357	1.244	0.058	0.095	0.007 - 1.004
	No	------	------	------	------	
Uses psychotropic drugs	Yes	1.716	1.042	0.100	5.561	0.766 - 48.862
	No	------	------	------	------	
Uses methylphenidate	Yes	15.957	------	0.994	------	0.000 - -----
	No	------	------	------	------	
Uses any stimulant medication with a medical prescription	Yes	13.013	------	0.995	------	0.000 - -----
	No	------	------	------	------	
Has a family history of mental disorder (father)	Yes	0.081	0.957	0.932	1.085	0.172 - 8.013
	No	------	------	------	------	
Performes physical activities	Yes	-1.543	0.793	0.052	0.214	0.039 - 0.930
	No	------	------	------	------	

*OR - Odds Ratio (Razão de Chances); IC - Intervalo de Confiança.*

Based on data obtained through the MINI - Module C, it was observed that 42.1% of undergraduate students were not at risk of suicide, while 23.9% were classified as low risk, 10.1% as moderate risk, and 23.9% as high risk. Logistic regression analysis identified four independent variables that contributed significantly to the predictive model. The factor with the greatest impact was the use of medications without a prescription, with an Odds Ratio (OR) of 10.537, followed by a previous diagnosis of a mental disorder (OR=10.234). These results indicate that undergraduate students who used medications without medical advice were 10.537 times more likely to be at risk of suicide, while those diagnosed with a mental disorder were 10.234 times more likely. Furthermore, the absence of religious ties (answer “none”) was associated with an 8.871-fold increase in the risk of suicide, and alcohol consumption was associated with a 4.495-fold greater chance of presenting this risk (Table 2).

**Table 2:** Logistic regression model adjusted for suicide risk in undergraduate students from different teaching units of a public university (N=190), Ribeirão Preto, São Paulo, Brazil, 2023

Variables		Coefficient	Standard error	Value *p*	OR^*^	95%CI^*^ (OR)
Intercept	-----------------	-9.401	3.760	0.012	0.000	0.000 - 0.64
Sexual orientation	Bissexual	3.781	2.398	0.115	43.849	0.444 - 9216.271
	Homossexual	3.253	2.646	0.219	25.856	0.157 - 8555.160
	Heterossexual	3.338	2.391	0.163	28.173	0.305 - 6218.043
	I do not want to say	2.915	3.683	0.429	18.440	0.024 - 46633.970
	Other	24.412	1903.624	0.990	-------	---------- - ---------
Family income	Greater than 1 to 3 minimum wages	2.299	2.018	0.255	9.962	0.210 - 805.067
	Greater than 3 to 5 minimum wages	3.220	2.066	0.119	25.020	0.505 - 2223.760
	Greater than 5 minimum wages	2.880	2.095	0.169	17.813	0.355 - 1694.190
	Less than 1 minimum wage	22.186	7093.070	0.998	-------	--------- - ---------
	No income	22.908	4435.366	0.996	-------	--------- - ---------
	I do not know	5.097	2.973	0.087	163.513	1.015-337769.400
	I prefer not to answer	4.627	2.848	0.104	102.173	0.712 - 54002.640
Religion	Spiritualist	0.737	1.236	0.551	2.089	0.190 - 26.306
	Evangelical	-0.273	1.522	0.858	0.761	0.021 - 12.249
	Others	1.912	1.258	0.129	6.768	0.614 - 94.333
	None	2.183	1.011	0.031	8.871	1.395 - 78.008
Uses any medication without a prescription (during the pandemic)	Yes	2.355	1.017	0.021	10.537	1.649 - 95.088
	No	------	------	------	------	------------------
Has a history of illness	Yes	1.635	0.878	0.062	5.131	1.019 - 34.288
	No	-------	------	------	------	------------------
Feels satisfied with life when accessed social media	Yes	-0.553	0.601	0.357	0.575	0.172 - 1.868
	No	-------	------	------	------	-----------------
Has a diagnosis of a mental disorder	Yes	-0.941	0.937	0.315	0.390	0.058 - 2.393
	No	------	------	------	-------	-----------------
Has a diagnosis of mental disorder	Yes	2.326	1.069	0.030	10.234	1.409 - 101.087
	No	------	------	------	-------	--------------------
Uses tobacco	Cigarette	2.386	1.738	0.170	10.868	0.619 - 734.452
Consumes alcohol	Yes	1.503	0.675	0.026	4.495	1.243 - 18.224
	No	-------	-------	-------	-------	-----------------
Uses illicit drugs	Yes	-0.828	0.945	0.381	0.437	0.063 - 2.731
	No	-------	-------	-------	-------	----------------
Currently, they use psychotropic drugs	Yes	-0.104	0.939	0.912	0.902	0.131 - 5.673
	No	------	------	-------	-------	-----------------
If you use any psychostimulant medication, use it with a doctor’s prescription.	Yes	0.845	1.236	0.495	2.327	0.227 - 32.103
	No	--------	-------	-------	-------	------------------

*OR - Odds Ratio; CI - Confidence Interval.*

It was found that 73.7% of students reported using stimulants. Bivariate analysis revealed statistically significant associations between this behavior and variables such as gender (p=0.028), perceived academic impairment during the COVID-19 pandemic (p=0.006), family history of cancer (p=0.023), psychological counseling (p=0.038), and alcohol consumption (p=0.008). To control for potential confounders, these variables were included in the multiple logistic regression model. However, after adjustment, none of them remained statistically associated with stimulant use (Table 3).

**Table 3:** Logistic regression model adjusted for stimulant use among undergraduate students from different teaching units of a public university (N=190), Ribeirão Preto, São Paulo, Brazil, 2023

Variables		Coefficient	Standard error	Value *p*	OR^*^	95%CI^*^ (OR)
Intercept	------------	-1.624	0.738	0.028	0.197	0.041 - 0.772
Gender	Male	0.185	0.613	0.763	1.203	0.333 - 3.844
	Female	------	------	------	------	
Suffered some loss (pandemic)	Yes	-0.583	0.640	0.362	0.558	0.164 - 2.101
	No	------	------	------	------	
Has a family history (cancer)	Yes	-0.449	0.514	0.383	0.638	0.230 - 1.770
	No	------	------	------	------	
Has psychological support	Yes	0.849	0.541	0.117	2.336	0.827 - 7.104
	No	------	------	------	------	
Consumes alcohol	Yes	-0.131	0.593	0.825	0.877	0.285 - 3.049
	No	------	------	------	------	

*OR - Odds Ratio; CI - Confidence Interval.*

## DISCUSSION

In this study, it was observed that 71.6% of participants tested positive for CMDs, a higher prevalence than that reported in previous studies, which indicate incidences between 50% and 66.1% in this population^(17-19)^, demonstrating the high vulnerability of students to CMDs. Furthermore, a higher prevalence of CMDs was found among female participants, in agreement with the literature^(20)^.

The high prevalence of CMDs in students is worrying, with a negative impact on quality of life, social relationships with friends and family, and academic performance, in addition to causing harm to academic performance^(17)^. Given this scenario, it is crucial to develop effective strategies aimed at preventing mental disorders and promoting mental health in this population. Such actions should include early screening and continuous monitoring, considering undergraduate students’ particularities and specific needs. The literature indicates that well-structured interventions can contribute to improving students’ physical, emotional, and psychological well-being^(17,21)^.

Logistic regression analysis showed that students who reported experiencing some type of harm during the pandemic were more likely to test positive for CMDs. The COVID-19 pandemic has brought significant changes to education systems, with direct consequences for students, such as the interruption of in-person activities for approximately 91% of students worldwide^(22)^. In the Brazilian context, a study with undergraduate students found that the worst mental health indicators were associated with conditions such as total social isolation, reduced income, increased alcohol consumption, and the direct impact of the pandemic on daily routines^(23)^.

In this sample, it was found that feeling satisfied with one’s life when using social media acted as a protective factor against CMDs. Although there is a gap in the literature regarding specific research on this variable in undergraduate students, studies conducted with graduate students indicate that the search for new forms of social interaction can be a compensatory strategy in the face of decreased subjective well-being. The use of social media, in this context, has proven to be an important tool in coping with physical isolation, reducing unpleasant psychological sensations, and promoting self-motivation^(24)^. In this regard, these platforms can favor the establishment of interpersonal bonds^(25)^.

It is important to highlight that, although social media can have positive effects under certain conditions, studies have shown that its excessive use is often associated with negative mental health outcomes, including symptoms of depression, anxiety, self-harm, body image dissatisfaction, eating disorders, sleep disorders, and reduced self-esteem, especially among young people^(26,27)^, including suicide risk. Brailovskaia *et al*.^(28)^ highlighted that this pattern of use is correlated with high suicide rates, especially in young and university populations.

In this study, 58% of participants were found to have some level of suicide risk, with 23.9% being classified as high risk. Global estimates indicate that more than 800,000 people die by suicide each year, making it the second leading cause of death among young people aged 15 to 29^(29)^. In Brazil, the scenario is also worrying, as suicide is the fourth leading cause of death in this age group, with approximately 13,523 deaths recorded in 2019^(30)^. Although the data in this research predate the COVID-19 pandemic, recent studies point to an increase in suicide risk among undergraduate students also during the pandemic and post-pandemic periods, which reinforces the relevance of the findings^(31,32)^.

Considering that the 15-29 age group is recognized as one of the most vulnerable to suicide, it is noteworthy that, in this study, the majority of participants (63.2%) were between 20 and 24 years old, which may, in part, explain the high proportion of undergraduate students at high risk of suicide. Given this reality, it is essential that higher education institutions implement prevention and care strategies, focusing on student support, mental health promotion, and preventing suicidal behavior^(33,34)^.

The data from this study indicated that the absence of religious affiliation was associated with an increased risk of suicide among participants. The literature suggests that religiosity can play a significant role in the lives of individuals experiencing suffering, offering meaning, hope, and a positive outlook in the face of adversity and moments of despair^(35)^.

National and international studies have also shown a higher prevalence of suicidal risk among individuals who have no religious affiliation^(36,37)^. A recent meta-analysis reinforces the hypothesis that religiosity can act as a protective factor against suicidal behavior. However, it highlights that the impact of religion on such behavior varies significantly depending on the cultural and religious contexts in which individuals are inserted^(38)^.

In this study, it was observed that the use of non-prescription medications was associated with a higher likelihood of suicide risk among participants. Supporting this finding, a recent study conducted with undergraduate students in Iraq found that individuals with and without a history of self-medication had high rates of suicidal ideation, although the prevalence was slightly higher among those who reported self-medication (66.7% versus 64.7%)^(39)^.

A study that investigated the relationship between self-medication and poisoning, based on data from the Santa Catarina Toxicological Information and Assistance Center, between 2014 and 2020, identified 683 cases of self-medication, with a higher prevalence in the 20-29 age group. Among the most commonly used drugs, anxiolytics stood out^(40)^. The literature indicates that this association may be due to the fact that anxiolytics are a means of execution or because their inappropriate use has the potential to cause suicidal behavior^(41)^. Furthermore, other research has shown that the use of antipsychotics and the practice of polypharmacy can also act as predictors of suicidal behavior^(42)^.

This study found that students previously diagnosed with a mental disorder were more likely to be at risk of suicide. A similar finding was observed in a study conducted with 496 undergraduate students in Iraq, which showed that a personal history of mental disorder acted as a significant predictor of suicidal ideation^(39)^, supporting the results of the present investigation.

Another study showed that students diagnosed with a mental disorder had a higher risk of suicide. The authors emphasize the importance of expanding the approach to this issue, with a view to improving undergraduate students’ quality of life. The study also reinforces the need to implement actions to promote mental health and prevent suicidal behavior by strengthening institutional strategies to support students in the academic environment^(43)^.

In this study, it was observed that alcohol consumption was associated with a higher likelihood of suicide risk among participants. This finding is supported by the literature, which indicates that the concomitant consumption of alcohol, tobacco, and other psychoactive substances is linked to the worsening of depressive symptoms in young people aged 15 to 30, which, in turn, contributes to the increased risk of suicide in this age group^(44)^.

The literature also indicates a greater chance of suicidal ideation among individuals who consume alcohol^(36,37)^. In the university context, stressful situations experienced by students, including difficulties in social adaptation and separation from family life, can encourage the abusive consumption of alcoholic beverages^(45)^. Thus, Cremasco and Baptista^(46)^ recommend, as a prevention strategy, seeking professional help when faced with warning signs, such as persistent feelings of anguish, anxiety or sadness, which can evolve into suicidal ideation.

This study revealed a high prevalence of stimulant use among participants, totaling 73.7%. Literature reinforces the importance of preventive measures in the university environment, warning of the risks associated with the indiscriminate use of these substances. It also emphasizes the need to implement educational programs and behavioral strategies aimed at managing academic stress, improving cognitive performance, and promoting students’ quality of life. Such interventions should include, among other measures, the inclusion of physical and recreational activities as a way to promote students’ physical and mental well-being^(47)^.

Logistic regression analysis indicated no statistically significant association between stimulant use and the variables investigated. However, a higher prevalence of stimulant use was observed among students who reported academic impairment. A study indicates that many undergraduate students turn to stimulants in the hope of accelerating the learning process, avoiding the need for continuous and regular study^(48)^. These drugs are often used to increase concentration, reduce drowsiness and enhance memory^(12)^. However, prolonged use of stimulants can cause changes in the noradrenergic and dopaminergic pathways, which can increase the risk of developing anxiety disorders and chemical dependency^(49)^.

It is also noteworthy that there was a higher prevalence of stimulant use among students who reported alcohol consumption. The literature points to the high frequency of concomitant alcohol and stimulant use among undergraduate students, a behavior often associated with the desire to explore the autonomy gained upon entering university as well as the search for new experiences typical of this transition period^(50)^.

### Study limitations

The results of this study must be interpreted in light of several methodological limitations. First, the cross-sectional design makes it impossible to infer causal relationships between the variables analyzed. Furthermore, there is the possibility of selection bias, since the sample consisted of volunteer participants, who may be more interested in or concerned about mental health issues. The use of self-administered questionnaires should also be considered, as they are subject to recall errors and response bias, especially regarding sensitive information such as substance use and symptoms of psychological distress, which respondents may be hesitant to report accurately.

### Contributions to nursing, health or public policy

This study makes a significant contribution to understanding the factors associated with stimulant use, suicide risk, and CMDs among undergraduate students, offering new insights for nursing practice and academic mental health interventions. It also fills a significant gap in knowledge on the topic, as although existing literature addresses individual aspects of mental health and substance use among students, there is a lack of studies that consider these factors comprehensively. Therefore, the findings provide a solid foundation for proposing policies and support programs focused on student mental health, focusing on the promotion and implementation of screening strategies, psychological support, and preventive education, which are essential for promoting undergraduate students’ quality of life and academic performance.

## CONCLUSIONS

Quantitative research revealed a high prevalence of CMDs among undergraduate students, suggesting that many of them face high levels of psychological distress. The likelihood of CMDs occurring was higher among those who reported experiencing some type of loss during the pandemic. On the other hand, perceived satisfaction with one’s life when using social media proved to be a protective factor against the development of these disorders.

The high prevalence of high suicide risk identified among students is particularly concerning and has been associated with factors such as lack of religion, use of over-the-counter medications, diagnosis of a mental disorder, and alcohol consumption. These associations reinforce the multifactorial nature of suicide risk, indicating the need for integrated and interdisciplinary approaches to its prevention.

Furthermore, the high prevalence of stimulant consumption suggests that these substances are commonly used in the academic environment, and the lack of direct association with the variables analyzed points to the need for further studies that consider other variables that may be influencing the use of these substances.

These findings highlight the high vulnerability of undergraduate students to suicide risk, stimulant use, and CMDs, highlighting the urgent need for integrated interventions that address this complex issue. Implementing strategies focused on self-care, psychosocial support, mental health education, and developing institutional policies sensitive to this population’s needs are essential to address these challenges and promote undergraduate well-being throughout their academic education.

## Data Availability

The research data are available within the article.
