# Supplementary material for: Factors associated with common mental disorders, suicidal risk, and stimulant use in college students
Source: Rev Bras Enferm. 2025 Dec 8;78(6):e20240501. doi: 10.1590/0034-7167-2024-0501 (PMC12707613; doi:10.1590/0034-7167-2024-0501)
Supplement: Translation

# Translation: Fatores associados aos transtornos mentais comuns, risco suicida e uso de estimulantes em estudantes universitários

**DOI:** 10.1590/0034-7167-2024-0501pt

**Authors:** Gisele Carolina Bianchi, Adriana Inocenti Miasso

**Affiliations:** I Universidade de São Paulo. Ribeirão Preto, São Paulo, Brasil

**Keywords:** Estudantes, Universidades, Transtornos Mentais, Suicídio, Psicotrópicos.

## Abstract

**Objetivos:**

identificar fatores associados a transtornos mentais comuns, risco suicida e uso de estimulantes em universitários.

**Métodos:**

estudo quantitativo, transversal, correlacional analítico, com amostra de 190 estudantes de uma universidade pública. Para coleta de dados, foram utilizados instrumentos validados e, para análise, modelos de regressão logística.

**Resultados:**

constatou-se que 71,6% dos participantes foram positivos para transtornos mentais comuns. Ter sofrido prejuízos na pandemia foi fator de risco para transtornos mentais comuns, e se sentir satisfeito com sua vida ao acessar as redes sociais foi fator de proteção. Verificou-se que 23,9% apresentaram alto risco suicida, maiores chances de risco daqueles sem religião, com uso de medicamentos sem prescrição, diagnóstico de transtorno mental e consumo de álcool. Ainda, 73,7% dos participantes utilizavam estimulantes. O uso de estimulantes não apresentou relação significativa com as variáveis analisadas.

**Conclusões:**

esta pesquisa contribui com evidências para ações de promoção e prevenção em saúde mental acadêmica.

## INTRODUÇÃO

O ingresso na universidade pode predispor os estudantes a maior vulnerabilidade à impaciência, irritabilidade, ansiedade e estresse, com potencial para transtornos mentais^(1)^. A pandemia de COVID-19 alterou a dinâmica acadêmica, com suspensão de atividades presenciais, estágios e adoção do ensino remoto, ocasionando impacto na qualidade da formação discente^(2)^. Essas mudanças ampliaram entre universitários sentimentos de angústia, além de sintomas ansiosos e depressivos^(3)^.

Pesquisa revela prevalência de 69,8% de transtornos mentais comuns (TMCs) em universitários após início da pandemia^(4)^.

Os TMCs correspondem a um conjunto de sintomas não psicóticos, como de ansiedade, depressão, insônia, fadiga, dores no corpo, irritabilidade, lapsos de memória, dificuldade de concentração e manifestações somáticas inespecíficas, que causam sofrimento psíquico e prejuízo funcional, mas não atendem perfeitamente aos critérios diagnósticos de transtornos mentais graves^(5)^.

Estudo aponta que o desenvolvimento de TMCs em estudantes pode estar associado ao sedentarismo, uso de tabaco e de substâncias atribuídas à melhoria do desempenho cognitivo, além de sentimentos de tristeza, anedonia, ansiedade, sintomas depressivos e ideação suicida^(6)^.

O risco suicida pode ser compreendido como a possibilidade de que pensamentos autodestrutivos evoluam para ações concretas, resultando em morte autoprovocada^(7)^. Trata-se de um fenômeno complexo, influenciado por uma variedade de determinantes, como fatores sociodemográficos, condições econômicas e aspectos relacionados à saúde, os quais afetam tanto a população em geral quanto grupos mais vulneráveis^(8)^, como os estudantes universitários. Estudos evidenciam que a presença de manifestações prévias de sofrimento psíquico, um quadro comum entre graduandos, contribui significativamente para maior suscetibilidade ao comportamento suicida^(9,10)^.

Diante das intensas demandas cognitivas e dos altos níveis de estresse vivenciados no ambiente universitário, há a possibilidade de que estudantes de graduação recorram a diferentes estratégias na tentativa de minimizar a sobrecarga emocional. Entre essas estratégias, destaca-se o uso de substâncias estimulantes, que atuam no sistema nervoso central promovendo aumento do estado de alerta, melhora da concentração, elevação da motivação e, em alguns casos, efeitos antidepressivos. Essas substâncias são frequentemente associadas à potencial melhoria do desempenho cognitivo e à regulação do humor^(11)^.

Pesquisa realizada com estudantes universitários revelou que 53,7% dos participantes relataram uso de estimulantes, sendo o cloridrato de metilfenidato, as bebidas energéticas e as anfetaminas os mais frequentemente mencionados. Os principais motivos apontados para esse consumo incluíram a busca por melhora das capacidades cognitivas, bem como a obtenção de um aprendizado mais rápido e eficaz^(12)^.

A utilização prolongada de substâncias estimulantes por estudantes universitários tem sido associada a diversos efeitos adversos, alguns dos quais podem ser permanentes. Entre os prejuízos identificados, destaca-se a possibilidade de agravamento de estados ansiosos, inicialmente fisiológicos, que podem evoluir para quadros patológicos mais severos, incluindo o aumento do risco de comportamento suicida. A falta de conhecimento sobre o uso dessas substâncias e os riscos associados constituem importante lacuna de conhecimento^(13)^.

Apesar da relevância dos TMCs, da utilização de estimulantes e do risco de suicídio entre estudantes universitários, observa-se uma lacuna na literatura quanto à investigação simultânea desses desfechos em amostras provenientes de instituições públicas de ensino superior. Aprofundar a compreensão sobre os fatores que contribuem para essas condições é fundamental para subsidiar estratégias de apoio mais eficazes e intervenções específicas, visando atenuar o sofrimento psíquico estudantil e fomentar um ambiente acadêmico mais saudável, acolhedor e sustentável.

## OBJETIVOS

Investigar os fatores associados aos TMCs, ao risco de suicídio e ao uso de estimulantes entre estudantes de uma universidade pública do estado de São Paulo, Brasil.

## MÉTODOS

### Aspectos éticos

Este estudo obteve aprovação do Comitê de Ética em Pesquisa (Certificado de Apresentação para Apreciação Ética n^o^ 55125921.2.0000.5393), seguindo as diretrizes estabelecidas pela Resolução nº 466, de 12 de dezembro de 2012, do Conselho Nacional de Saúde, que regulamenta pesquisas envolvendo seres humanos. Todos os participantes assinaram o Termo de Consentimento Livre e Esclarecido (TCLE).

Este artigo é derivado da dissertação de mestrado intitulada “Transtornos mentais comuns, risco de suicídio e uso de estimulantes em estudantes de uma universidade pública”^(14)^.

### Desenho, período e local do estudo

Estudo com abordagem quantitativa de delineamento transversal e natureza correlacional analítico, realizado de março a julho de 2023 em unidades de ensino superior situadas em um *campus* de uma universidade pública no estado de São Paulo, Brasil. A condução da pesquisa foi embasada pela ferramenta *STrengthening the Reporting of OBservational studies in Epidemiology*.

### População e amostra: critérios de inclusão e exclusão

A amostra, selecionada por conveniência, compreendeu 190 estudantes matriculados nos cursos de graduação do *campus* universitário investigado. Foram incluídos estudantes com 18 anos ou mais e que estar regularmente matriculados nos cursos de graduação da instituição. Foram excluídos aqueles que preencheram o instrumento de forma incompleta, de modo a inviabilizar a análise das três variáveis desfechos do estudo: TMC, risco de suicídio e uso de estimulantes.

### Protocolo do estudo

Para a coleta de dados, foi enviado um *e-mail* a todas as Comissões de Graduação das unidades do *campus* investigado, solicitando o encaminhamento da carta convite aos estudantes. Dessa forma, todos os alunos de graduação receberam um convite formal individual por meio das Comissões de Graduação via *e-mail* institucional. A carta apresentou o título da pesquisa, seu objetivo, a justificativa e as possíveis contribuições do estudo, incluindo ao final o *link* para acesso à plataforma *Google Forms* ^®^.

Os estudantes que consentiram em participar da pesquisa acessaram o *link* disponibilizado, no qual se encontraram o TCLE e os instrumentos de coleta de dados. Ao concordar e assinar eletronicamente o TCLE, foram encaminhados para o preenchimento dos questionários. O período para resposta foi estabelecido em 60 dias a partir do envio da carta convite, após o qual o recebimento dos instrumentos foi oficialmente encerrado.

Foram empregados três instrumentos para coleta dos dados: 1 - Questionário elaborado pelas pesquisadoras, abrangendo informações sociodemográficas, econômicas, acadêmicas, sobre histórico de saúde e uso de estimulantes, que passou por avaliação de forma e conteúdo por especialistas na área; 2 - *Self-Reporting Questionnaire*-20, contendo 20 questões para rastreamento de TMC, validado para uso no Brasil^(15)^. Neste estudo, adotaram-se os pontos de corte estabelecidos na validação: caso positivo para mulheres que assinalaram “sim” em oito ou mais itens, e para homens, com seis ou mais respostas afirmativas; 3 - *Mini International Neuropsychiatric Interview* (MINI) - um instrumento diagnóstico breve alinhado aos critérios do DSM-III-R/IV e da Classificação Internacional de Doenças, 10ª revisão, reconhecido por sua confiabilidade e validade internacional^(16)^. O instrumento é organizado por módulos diagnósticos independentes, e as respostas consistem em uma variável dicotômica de “sim” ou “não”^(16)^. Organizado em módulos diagnósticos independentes, o MINI utiliza respostas dicotômicas (sim/não), sendo que, para os objetivos deste estudo, foi aplicado especificamente o módulo C, referente ao risco de suicídio.

### Análise dos resultados e estatística

A análise dos dados foi realizada por meio da abordagem quantitativa. As respostas obtidas por meio da plataforma *Google Forms* ^®^ foram exportadas para uma planilha e, posteriormente, transferidas para o *software Statistical Package for the Social Sciences*, versão 21.5.

Inicialmente, foram realizadas análises univariadas utilizando o teste qui-quadrado. Nos casos em que as categorias apresentaram frequência inferior a cinco participantes, aplicou-se o teste exato de Fisher. Para avaliar a influência das variáveis independentes sobre os desfechos do estudo, foram desenvolvidos modelos de regressão logística multivariada. Integraram o modelo as variáveis independentes que apresentaram valor de p inferior a 0,05 na análise univariada. Foram consideradas estatisticamente significativas as associações com p<0,05.

## RESULTADOS

Participaram desta pesquisa 190 estudantes universitários. A maioria dos participantes informou não ter filhos (98,4%), não ter companheiro (92,5%), ser procedente do estado de São Paulo (82,6%), possuir cor branca (77,5%), ser do gênero feminino (71,8%), estar na faixa etária de 20 a 24 anos (63,2%) e possuir orientação heterossexual (51,6%).

Constatou-se que 71,6% dos participantes foram positivos para TMCs. O modelo de regressão logística evidenciou que participantes que afirmaram ter sofrido algum prejuízo na pandemia tiveram 10,278 vezes mais chance de serem positivos para TMC (OR=10.278; IC=2.301 - 57.446; p=0.004). Sentir-se satisfeito com a própria vida ao utilizar redes sociais se mostrou um fator protetor contra TMC (OR=0.071; IC=0.013 - 0.286; p=0.001) (Tabela 1).

**Tabela 1:** Modelo de regressão logística ajustado para transtornos mentais comuns em graduandos de diferentes unidades de ensino de uma universidade pública (N=190), Ribeirão Preto, São Paulo, Brasil, 2023

Variáveis		Coeficiente	Erro padrão	Valor de *p*	OR^*^	IC^*^ (OR) 95%
Intercepto	-----	2,115	1,145	0,065	8,287	0,959 - 91,298
Sofreu algum prejuízo na pandemia	Sim	2,330	0,807	0,004	10,278	2,301 - 57,446
	Não	------	------	------	------	
Testou positivo (COVID-19)	Sim	-1,142	0,695	0,100	0,319	0,075 - 1,191
	Não	------	------	------	------	
Possui histórico de doenças	Sim	0,124	0,857	0,885	1,132	0,202 - 6,081
	Não	------	------	------	------	
Sente-se satisfeito com sua vida ao acessar as redes sociais	Sim	-2,642	0,777	0,001	0,071	0,013 - 0,286
	Não	------	------	------	------	
Tem algum diagnóstico de transtorno mental	Sim	0,680	1,097	0,535	1,975	0,230 - 18,152
	Não	------	------	------	------	
Possui diagnóstico de transtorno mental	Sim	-0,026	0,917	0,977	0,974	0,160 - 6,200
	Não	------	------	------	------	
Usa tabaco (POD)	Sim	-2,357	1,244	0,058	0,095	0,007 - 1,004
	Não	------	------	------	------	
Usa psicofármacos	Sim	1,716	1,042	0,100	5,561	0,766 - 48,862
	Não	------	------	------	------	
Usa metilfenidato	Sim	15,957	------	0,994	------	0,000 - -----
	Não	------	------	------	------	
Utiliza algum medicamento estimulante com prescrição médica	Sim	13,013	------	0,995	------	0,000 - -----
	Não	------	------	------	------	
Possui histórico familiar de transtorno mental (pai)	Sim	0,081	0,957	0,932	1,085	0,172 - 8,013
	Não	------	------	------	------	
Realiza atividades físicas	Sim	-1,543	0,793	0,052	0,214	0,039 - 0,930
	Não	------	------	------	------	

*OR - Odds Ratio (Razão de Chances); IC - Intervalo de Confiança.*

Com base nos dados obtidos por meio do MINI - módulo C, observou-se que 42,1% dos graduandos não apresentaram risco de suicídio, enquanto 23,9% foram classificados com risco baixo, 10,1%, com risco moderado e 23,9%, com risco alto. A análise de regressão logística identificou quatro variáveis independentes que contribuíram significativamente para o modelo preditivo. O fator de maior impacto foi o uso de medicamentos sem prescrição médica, com um *Odds Ratio* (OR) de 10,537, seguido pelo diagnóstico prévio de transtorno mental (OR=10,234). Esses resultados indicam que graduandos que utilizaram medicamentos sem orientação médica apresentaram uma chance 10,537 vezes maior de estar em risco de suicídio, enquanto aqueles com diagnóstico de transtorno mental apresentaram uma chance 10,234 vezes maior. Além disso, a ausência de vínculo religioso (resposta “nenhuma”) esteve associada a um aumento de 8,871 vezes no risco de suicídio, e o uso de álcool foi associado a uma chance 4,495 vezes maior de apresentar esse risco (Tabela 2).

**Tabela 2:** Modelo de regressão logística ajustado para risco de suicídio em graduandos de diferentes unidades de ensino de uma universidade pública (N=190), Ribeirão Preto, São Paulo, Brasil, 2023

Variáveis		Coeficiente	Erro padrão	Valor de *p*	OR^*^	IC^*^ (OR) 95%
Intercepto	-----------------	-9,401	3,760	0,012	0,000	0,000 - 0,64
Orientação sexual	Bissexual	3,781	2,398	0,115	43,849	0,444 - 9216,271
	Homossexual	3,253	2,646	0,219	25,856	0,157 - 8555,160
	Heterossexual	3,338	2,391	0,163	28,173	0,305 - 6218,043
	Não quero dizer	2,915	3,683	0,429	18,440	0,024 - 46633,970
	Outros	24,412	1903,624	0,990	-------	---------- - ---------
Renda familiar	Maior que 1 a 3 salários mínimos	2,299	2,018	0,255	9,962	0,210 - 805,067
	Maior que 3 a 5 salários mínimos	3,220	2,066	0,119	25,020	0,505 - 2223,760
	Maior que 5 salários mínimos	2,880	2,095	0,169	17,813	0,355 - 1694,190
	Menor que 1 salário mínimo	22,186	7093,070	0,998	-------	--------- - ---------
	Sem renda	22,908	4435,366	0,996	-------	--------- - ---------
	Não sei	5,097	2,973	0,087	163,513	1,015-337769,400
	Prefiro não responder	4,627	2,848	0,104	102,173	0,712 - 54002,640
Religião	Espírita	0,737	1,236	0,551	2,089	0,190 - 26,306
	Evangélico	-0,273	1,522	0,858	0,761	0,021 - 12,249
	Outras	1,912	1,258	0,129	6,768	0,614 - 94,333
	Nenhuma	2,183	1,011	0,031	8,871	1,395 - 78,008
Utilizou algum medicamento sem prescrição médica (durante a pandemia)	Sim	2,355	1,017	0,021	10,537	1,649 - 95,088
	Não	------	------	------	------	------------------
Possui histórico de doenças	Sim	1,635	0,878	0,062	5,131	1,019 - 34,288
	Não	-------	------	------	------	------------------
Você se sente satisfeito com sua vida quando acessa as redes sociais	Sim	-0,553	0,601	0,357	0,575	0,172 - 1,868
	Não	-------	------	------	------	-----------------
Possui diagnóstico de algum transtorno mental	Sim	-0,941	0,937	0,315	0,390	0,058 - 2,393
	Não	------	------	------	-------	-----------------
Possui diagnóstico de transtorno mental	Sim	2,326	1,069	0,030	10,234	1,409 - 101,087
	Não	------	------	------	-------	--------------------
Usa tabaco	Cigarro	2,386	1,738	0,170	10,868	0,619 - 734,452
Usa álcool	Sim	1,503	0,675	0,026	4,495	1,243 - 18,224
	Não	-------	-------	-------	-------	-----------------
Usa drogas ilícitas	Sim	-0,828	0,945	0,381	0,437	0,063 - 2,731
	Não	-------	-------	-------	-------	----------------
Atualmente, faz uso de psicofármacos	Sim	-0,104	0,939	0,912	0,902	0,131 - 5,673
	Não	------	------	-------	-------	-----------------
Se utiliza algum medicamento psicoestimulante, o uso é com prescrição médica	Sim	0,845	1,236	0,495	2,327	0,227 - 32,103
	Não	--------	-------	-------	-------	------------------

*OR - Odds Ratio (Razão de Chances); IC - Intervalo de Confiança.*

Identificou-se que 73,7% dos estudantes relataram o uso de estimulantes. A análise bivariada revelou associações estatisticamente significativas entre esse comportamento e variáveis como gênero (p=0,028), percepção de prejuízo acadêmico durante a pandemia de COVID-19 (p=0,006), histórico familiar de câncer (p=0,023), acompanhamento psicológico (p=0,038) e consumo de álcool (p=0,008). Com o objetivo de controlar potenciais fatores de confusão, essas variáveis foram incluídas no modelo de regressão logística múltipla. Contudo, após o ajuste, nenhuma delas permaneceu estatisticamente associada ao uso de estimulantes (Tabela 3).

**Tabela 3:** Modelo de regressão logística ajustado para uso de estimulantes em graduandos de diferentes unidades de ensino de uma universidade pública (N=190), Ribeirão Preto, São Paulo, Brasil, 2023

Variáveis		Coeficiente	Erro padrão	Valor de *p*	OR^*^	IC^*^ (OR) 95%
Intercepto	------------	-1,624	0,738	0,028	0,197	0,041 - 0,772
Gênero	Masculino	0,185	0,613	0,763	1,203	0,333 - 3,844
	Feminino	------	------	------	------	
Sofreu algum prejuízo (pandemia)	Sim	-0,583	0,640	0,362	0,558	0,164 - 2,101
	Não	------	------	------	------	
Possui histórico familiar (câncer)	Sim	-0,449	0,514	0,383	0,638	0,230 - 1,770
	Não	------	------	------	------	
Possui acompanhamento psicológico	Sim	0,849	0,541	0,117	2,336	0,827 - 7,104
	Não	------	------	------	------	
Usa álcool	Sim	-0,131	0,593	0,825	0,877	0,285 - 3,049
	Não	------	------	------	------	

*OR - Odds Ratio (Razão de Chances); IC - Intervalo de Confiança.*

## DISCUSSÃO

Neste estudo, observou-se que 71,6% dos participantes apresentaram resultado positivo para TMCs, uma prevalência superior à relatada em estudos anteriores, que indicam incidências entre 50% e 66,1% nessa população^(17-19)^, mostrando alta vulnerabilidade dos estudantes aos TMCs. Ademais, verificou-se maior prevalência de TMCs entre as participantes do sexo feminino, em concordância com a literatura^(20)^.

A alta prevalência de TMCs em estudantes é preocupante, com impacto negativo na qualidade de vida, nas relações sociais com amigos, familiares e no âmbito acadêmico, além de gerar prejuízos no desempenho acadêmico^(17)^. Diante desse cenário, torna-se importante o desenvolvimento de estratégias eficazes voltadas à prevenção dos transtornos mentais e à promoção da saúde mental nessa população. Tais ações devem contemplar o rastreamento precoce e o monitoramento contínuo, considerando as particularidades e demandas específicas dos graduandos. A literatura aponta que intervenções bem estruturadas podem contribuir para a melhoria do bem-estar físico, emocional e psicológico dos estudantes^(17,21)^.

A análise por regressão logística mostrou que estudantes que relataram ter vivenciado algum tipo de prejuízo durante a pandemia apresentaram mais chance de serem positivos para TMCs. A pandemia de COVID-19 provocou mudanças relevantes nos sistemas educacionais, com consequências diretas para os estudantes, como a interrupção das atividades presenciais para aproximadamente 91% dos alunos em todo o mundo^(22)^. No contexto brasileiro, estudo com universitários identificou que os piores indicadores de saúde mental estiveram associados a condições como isolamento social total, redução da renda, aumento do consumo de álcool e impacto direto da pandemia na rotina diária^(23)^.

Verificou-se, nesta amostra, que sentir-se satisfeito com a própria vida ao utilizar redes sociais atuou como fator de proteção em relação aos TMCs. Embora haja uma lacuna na literatura quanto à investigação específica dessa variável em estudantes de graduação, estudos realizados com pós-graduandos indicam que a busca por novas formas de interação social pode ser uma estratégia compensatória diante da diminuição do bem-estar subjetivo. O uso de redes sociais, nesse contexto, tem se mostrado uma ferramenta importante no enfrentamento do isolamento físico, na redução de sensações psicológicas desagradáveis e na promoção da automotivação^(24)^. Nesse sentido, essas plataformas podem favorecer o estabelecimento de vínculos interpessoais^(25)^.

É importante destacar que, embora as redes sociais possam exercer efeitos positivos sob determinadas condições, estudos têm mostrado que seu uso excessivo está frequentemente relacionado a desfechos negativos em saúde mental, incluindo sintomas de depressão, ansiedade, comportamentos de automutilação, insatisfação com a imagem corporal, transtornos alimentares, distúrbios do sono e redução da autoestima, especialmente entre jovens^(26,27)^, incluindo risco de suicídio. Brailovskaia *et al*.^(28)^ destacam que esse padrão de uso está correlacionado a taxas elevadas de suicídio, sobretudo em populações jovens e universitárias.

Nesta pesquisa, verificou-se que 58% dos participantes apresentaram algum nível de risco de suicídio, com destaque para os 23,9% que foram classificados com risco alto. Estimativas globais indicam que mais de 800 mil pessoas morrem por suicídio a cada ano, sendo esta a segunda principal causa de morte entre jovens com idades entre 15 e 29 anos^(29)^. No Brasil, o cenário também é preocupante, pois o suicídio figura como a quarta principal causa de óbito nessa faixa etária, com aproximadamente 13.523 mortes registradas em 2019^(30)^. Embora os dados desta pesquisa sejam anteriores à pandemia de COVID-19, estudos recentes apontam aumento no risco suicida entre universitários também no período pandêmico e pós-pandêmico, o que reforça a relevância dos achados^(31,32)^.

Considerando que a faixa etária entre 15 e 29 anos é reconhecida como uma das mais vulneráveis ao suicídio, ressalta-se que, nesta pesquisa, a maioria dos participantes (63,2%) tinha entre 20 e 24 anos, o que pode, em parte, explicar a elevada proporção de graduandos com risco alto de suicídio. Diante dessa realidade, torna-se imprescindível que as instituições de ensino superior implementem estratégias de prevenção e cuidado, com foco no acolhimento estudantil, promoção da saúde mental e prevenção de comportamentos suicidas^(33,34)^.

Os dados desta pesquisa indicaram que a ausência de vínculo religioso esteve associada a um aumento no risco de suicídio entre os participantes. A literatura aponta que a religiosidade pode desempenhar um papel significativo na vida de indivíduos em situações de sofrimento, oferecendo sentido, esperança e uma perspectiva positiva diante de adversidades e momentos de desesperança^(35)^.

Estudos nacionais e internacionais também têm mostrado uma maior prevalência de risco suicida entre indivíduos que não possuem afiliação religiosa^(36,37)^. Meta-análise recente reforça a hipótese de que a religiosidade pode atuar como fator protetivo contra comportamentos suicidas. No entanto, ressalta que o impacto da religião sobre tais comportamentos apresenta variações importantes, dependendo dos contextos culturais e religiosos em que os indivíduos estão inseridos^(38)^.

Nesta pesquisa, observou-se que o uso de medicamentos sem prescrição médica esteve associado a uma maior probabilidade de risco de suicídio entre os participantes. Corroborando esse achado, estudo recente realizado com estudantes universitários no Iraque identificou que indivíduos com ou sem histórico de automedicação apresentaram taxas elevadas de ideação suicida, embora a prevalência tenha sido ligeiramente maior entre aqueles que relataram automedicação (66,7% *versus* 64,7%)^(39)^.

Estudo que investigou a relação entre automedicação e intoxicação, com base em dados do Centro de Informação e Assistência Toxicológica de Santa Catarina, entre os anos de 2014 e 2020, identificou 683 casos de automedicação, com maior prevalência na faixa etária de 20 a 29 anos. Entre os fármacos mais utilizados, destacaram-se os ansiolíticos^(40)^. A literatura aponta que essa referida associação pode ser tanto pelo fato de os ansiolíticos serem meio para execução quanto pelo seu uso inadequado ser potencial para comportamento suicida^(41)^. Além disso, outra pesquisa mostrou que o uso de antipsicóticos e a prática de polifarmácia também podem atuar como preditores de comportamento suicida^(42)^.

Verificou-se, nesta pesquisa, que estudantes diagnosticados previamente com transtorno mental apresentaram mais chance de risco suicida. Achado semelhante foi observado em estudo conduzido com 496 estudantes universitários no Iraque, o qual evidenciou que o histórico pessoal de transtorno mental atuou como preditor significativo para a ideação suicida^(39)^, corroborando os resultados da presente investigação.

Outro estudo mostrou que estudantes com diagnóstico de transtorno mental tiveram maior risco de suicídio. Os autores destacam a importância de ampliar a forma como essa questão é abordada, com vistas à melhoria da qualidade de vida dos universitários. O estudo também reforça a necessidade de implementar ações de promoção da saúde mental e prevenção do comportamento suicida, por meio do fortalecimento das estratégias institucionais de apoio aos alunos no ambiente acadêmico^(43)^.

Neste estudo, observou-se que o consumo de álcool esteve associado a maior probabilidade de risco de suicídio entre os participantes. Esse achado é respaldado pela literatura, que aponta que o uso concomitante de álcool, tabaco e outras substâncias psicoativas está relacionado ao agravamento de sintomas depressivos em jovens com idade entre 15 e 30 anos, o que, por sua vez, contribui para o aumento do risco de suicídio nesse grupo etário^(44)^.

A literatura também aponta mais chances de ideação suicida entre indivíduos que fazem uso de álcool^(36,37)^. No contexto universitário, situações estressoras vivenciadas pelos estudantes, incluindo as dificuldades de adaptação social e afastamento do convívio familiar, podem favorecer o consumo abusivo de bebidas alcoólicas^(45)^. Nesse sentido, Cremasco e Baptista^(46)^ recomendam, como estratégia de prevenção, a busca por ajuda profissional diante de sinais de alerta, como sentimentos persistentes de angústia, ansiedade ou tristeza, que podem evoluir para quadros de ideação suicida.

Esta pesquisa revelou uma elevada prevalência de uso de estimulantes entre os participantes, totalizando 73,7%. A literatura reforça a importância de ações preventivas no ambiente universitário, alertando para os riscos associados ao uso indiscriminado dessas substâncias. Ressalta-se, ainda, a necessidade da implementação de programas educacionais e estratégias comportamentais voltados à gestão do estresse acadêmico, à melhoria do desempenho cognitivo e à promoção da qualidade de vida dos estudantes. Tais intervenções devem contemplar, entre outras medidas, a inserção de atividades físicas e recreativas como forma de favorecer o bem-estar físico e mental dos estudantes^(47)^.

A análise por regressão logística indicou ausência de associação estatisticamente significativa entre o uso de estimulantes e as variáveis investigadas. No entanto, observou-se maior prevalência de consumo dessas substâncias entre os estudantes que mencionaram prejuízo acadêmico. Estudo indica que muitos universitários recorrem aos estimulantes na expectativa de acelerar o processo de aprendizagem, evitando a necessidade de estudos contínuos e regulares^(48)^. Esses fármacos são frequentemente utilizados com o intuito de aumentar a concentração, reduzir a sonolência e potencializar a memória^(12)^. Entretanto, o uso prolongado de estimulantes pode provocar alterações nas vias noradrenérgicas e dopaminérgicas, o que pode elevar o risco para o desenvolvimento de transtornos de ansiedade e dependência química^(49)^.

Destaca-se, ainda, que houve maior prevalência de uso de estimulantes entre os estudantes que relataram consumo de álcool. A literatura aponta para a elevada frequência do uso concomitante de álcool e estimulantes entre universitários, comportamento frequentemente associado ao desejo de explorar a autonomia adquirida com a entrada na universidade, bem como à busca por novas experiências típicas desse período de transição^(50)^.

### Limitações do estudo

Os resultados deste estudo devem ser interpretados à luz de algumas limitações metodológicas. Em primeiro lugar, o delineamento transversal impossibilita a inferência de relações causais entre as variáveis analisadas. Além disso, há a possibilidade de viés de seleção, uma vez que a amostra foi composta por participantes voluntários, os quais podem apresentar maior interesse ou preocupação com questões relacionadas à saúde mental. Também se deve considerar o uso de questionários autoadministrados, os quais estão sujeitos a erros de recordação e viés de resposta, especialmente em relação a informações sensíveis, como o uso de substâncias psicoativas e sintomas de sofrimento psíquico, que os respondentes podem hesitar em relatar com precisão.

### Contribuições para as áreas da enfermagem, saúde ou políticas públicas

Este estudo contribui de modo relevante para a compreensão dos fatores associados ao uso de estimulantes, risco suicida e TMCs entre graduandos, oferecendo novos *insights* para a prática de enfermagem e intervenções na saúde mental acadêmica. Preenche, ainda, uma lacuna significativa no conhecimento sobre a temática, pois embora a literatura existente aborde individualmente os aspectos da saúde mental e do uso de substâncias entre estudantes, há carência de estudos que considerem esses fatores de forma abrangente. Nessa direção, os achados fornecem uma base sólida para a proposição de políticas e programas de apoio voltados à saúde mental dos estudantes, com foco na promoção e implementação de estratégias de rastreio, suporte psicológico e educação preventiva, fundamentais para promoção da qualidade de vida e desempenho acadêmico dos graduandos.

## CONCLUSÕES

A pesquisa quantitativa evidenciou uma elevada prevalência de TMCs entre os universitários, sugerindo que muitos deles enfrentam níveis elevados de sofrimento psíquico. Observou-se que a probabilidade de ocorrência de TMCs foi maior entre aqueles que relataram ter experimentado algum tipo de prejuízo durante a pandemia. Por outro lado, a percepção de satisfação com a própria vida ao utilizar redes sociais se mostrou como um fator de proteção diante do desenvolvimento desses transtornos.

A prevalência elevada de risco alto de suicídio identificada entre os estudantes é especialmente preocupante e foi associada a fatores como ausência de religião, uso de medicamentos sem prescrição, diagnóstico de transtorno mental e consumo de álcool. Tais associações reforçam a natureza multifatorial do risco suicida, indicando a necessidade de abordagens integradas e interdisciplinares para sua prevenção.

Além disso, a prevalência elevada do consumo de estimulantes sugere que essas substâncias são comumente utilizadas no ambiente acadêmico, e a ausência de associação direta com as variáveis analisadas aponta para a necessidade de novos estudos que considerem outras variáveis que possam estar influenciando o uso dessas substâncias.

Esses achados evidenciam a alta vulnerabilidade dos graduandos ao risco de suicídio, uso de estimulantes e aos TMCs, evidenciando a urgência de intervenções integradas que abordem essa complexidade. A implementação de estratégias voltadas ao autocuidado, suporte psicossocial, educação em saúde mental e desenvolvimento de políticas institucionais sensíveis às demandas dessa população são fundamentais para enfrentar esses desafios e promover o bem-estar dos graduandos ao longo de sua formação acadêmica.

## Data Availability

Os dados da pesquisa estão disponíveis no corpo do artigo.
